# 

**Published:** 1858-03

**Authors:** 


☞ V. H. TALIAFERRO, M. D., a promising physician of this city, and a frequent and valuable contributor to this Journal, has been appointed Professor of Materia Medica and Therapeutics in the Oglethorpe Medical College at Savannah.

### Payments for the Journal.

W. H. Moore, N. C., 3d Vol.; M. D. Stribling, Ga., 3d Vol.; J. F. Woodbury, Ga., 3d Vol.; M. A. Leak, Ga., 3d Vol.; Jos. Michaux, Va., 2d and 3d Vol.; W. Burch, Ga., 2d and 3d Vol.: A. S. Mason, Ga., 3d Vol.; T. M. Darnall, Ga., 3d Vol.; T. A. Reines Ga., 3d Vol.

